# Impact of a chronic disease self-management program on health care utilization in rural communities: a retrospective cohort study using linked administrative data

**DOI:** 10.1186/1472-6963-14-198

**Published:** 2014-05-01

**Authors:** Susan B Jaglal, Sara JT Guilcher, Gillian Hawker, Wendy Lou, Nancy M Salbach, Michael Manno, Merrick Zwarenstein

**Affiliations:** 1 Women’s College Research Institute, Toronto, Ontario, Canada; 2 Department of Physical Therapy, Faculty of Medicine, University of Toronto, Toronto, Ontario, Canada; 3 Toronto Rehabilitation Institute-University Health Network, Toronto, Ontario, Canada; 4 Institute for Clinical Evaluative Sciences, Toronto, Ontario, Canada; 5 Institute of Health Policy, Management and Evaluation, University of Toronto, Toronto, Ontario, Canada; 6 Centre for Research on Inner City Health, St. Michael’s Hospital, Toronto, Ontario, Canada; 7 Department of Medicine, Women’s College Hospital, Toronto, Ontario, Canada; 8 Dalla Lana School of Public Health, University of Toronto, Health Sciences Building, Toronto, Ontario, Canada; 9 The Ontario HIV Treatment Network, Toronto, Ontario, Canada; 10 Centre for Studies in Family Medicine & Department of Family Medicine, Western University, London, Ontario, Canada

## Abstract

**Background:**

Internationally, chronic disease self-management programs (CDSMPs) have been widely promoted with the assumption that confident, knowledgeable patients practicing self-management behavior will experience improved health and utilize fewer healthcare resources. However, there is a paucity of published data supporting this claim and the majority of the evidence is based on self-report.

**Methods:**

We used a retrospective cohort study using linked administrative health data. Data from 104 tele-CDSMP participants from 13 rural and remote communities in the province of Ontario, Canada were linked to administrative databases containing emergency department (ED) and physician visits and hospitalizations. Patterns of health care utilization prior to and after participation in the tele-CDSMP were compared. Poisson Generalized Estimating Equations regression was used to examine the impact of the tele-CDSMP on health care utilization after adjusting for covariates.

**Results:**

There were no differences in patterns of health care utilization before and after participating in the tele-CDSMP. Among participants ≤ 66 years, however, there was a 34% increase in physician visits in the 12 months following the program (OR = 1.34, 95% CI 1.11-1.61) and a trend for decreased ED visits in those >66 years (OR = 0.59, 95% CI 0.33-1.06).

**Conclusions:**

This is the first study to examine health care use following participation in the CDSMP in a Canadian population and to use administrative data to measure health care utilization. Similar to other studies that used self-report measures to evaluate health care use we found no differences in health care utilization before and after participation in the CDSMP. Future research needs to confirm our findings and examine the impact of the CDSMP on health care utilization in different age groups to help to determine whether these interventions are more effective with select population groups.

## Background

Self-management programs are widely recommended to empower participants, increase their confidence, teach skills and techniques, and improve their interaction with the health care system to enable better management of chronic conditions [1]. A number of governments have adopted the Chronic Disease Self-Management Program (CDSMP), which was developed and validated by the Stanford Patient Education Research Centre as part of population-based long-term chronic disease management strategies [2,3]. For example, the Department of Health in the UK has invested $36 million (US) in the Expert Patient Programme (EPP), an adaptation of the CDSMP for delivery to 100,000 patients by 2012 [4]. In Australia, the CDSMP and other patient programs are being delivered through the Sharing Health Care Initiative, with large demonstration projects across a variety of settings [5]. In the US, the CDSMP has been implemented within a large health maintenance organization [6]. Most Canadian provinces and territories fund community-based programs for patients in self-management education using versions of the CDSMP [7]. There is significant interest in the potential for the CDSMP to improve health outcomes and reduce health care use, ultimately lowering health care costs and creating a healthier society [2,4,7].

One of the main tenets of the CDSMP is that confident, knowledgeable patients practicing self-management will experience improved health status and utilize fewer health care resources [8-10]. However, there is a paucity of data supporting the claim of reduced health care utilization. A recent meta-analysis by Brady and colleagues found that about half of the CDSMP studies reported some health care utilization data (number of physician visits, number of emergency department (ED) visits, number of times hospitalized, and number of days or nights in the hospital) and all but one used self-report measures [11]. Only Ritter et al [12], in a RCT of 216 subjects evaluating the CDSMP used computerized medical records from a health maintenance organization to determine health care utilization. The meta-analysis included 8,688 participants (2,902 enrolled in randomized controlled trials (RCTs) and 5,779 in longitudinal studies). Of the 23 studies, only 9 of them counted physician visits, 8 measured days hospitalized and 7 hospitalizations at 4 or 6 months post-intervention respectively. Only 4 studies measured health care utilization at 9 or 12 months. Overall, changes were minimal with no significant effect sizes at 4 or 6 months or 9 or12 months post-intervention for physician visits, ED visits or hospitalizations. There was a small but statistically significant decrease in days hospitalized, at 4 or 6 months that did not persist at 9 or 12 months. Recently, Ory and colleagues identified significant reductions in self-reported ED visits at 12 months which were not observed for self-reported hospitalizations [13]. Brady and colleagues suggested in their meta-analysis that administrative claims data would provide a more definitive examination of the impact of the CDSMP on health care utilization given that self-reported measures of health care utilization are subject to recall bias [14]. To date, no study has evaluated the impact of the CDSMP on health care utilization using administrative claims data.

We recently conducted an evaluation of a telehealth version of the Stanford chronic disease self-management program (tele-CDSMP) to determine if it would lead to improvements in self-efficacy, health behaviours, and health status for older adults with chronic disease living in rural and remote areas in Northern Ontario, Canada [15]. Participants had a self-reported physician diagnosis of chronic lung disease, heart disease, stroke, or chronic arthritis. Individuals with diabetes and hypertension were included if they also had one of the above diagnoses. The tele-CDSMP was an adaptation of the CDSMP delivered via telehealth. The lay leaders were recruited from five communities with populations greater than 40,000 inhabitants. At least 2 of the 3 lay leaders per team had one of the chronic diseases targeted in the study and the other was either a health care professional or other professional. The leaders linked via videoconferencing, using the Ontario Telemedicine Network infrastructure, to participants recruited from 13 communities ranging in population from approximately 1,400 to 18,000 inhabitants. The highly interactive course used the content of the CDSMP, and included a) guided mastery of skills through weekly action planning and feedback of progress, b) modeling of self-management behaviors and problem-solving strategies, and c) social persuasion through group support and guidance for individual self-management efforts [16]**].** The CDSMP taught the following content to participants in six weekly 2-hour sessions: how to develop an exercise program, cognitive symptom management, breathing exercises, problem solving, communication skills (with family, friends, and health care providers), use of medication, and dealing with the emotions of chronic illness (anger and depression). Participants experienced significant improvements in self-efficacy, a number of health behaviors (exercise, cognitive symptom management, communication with physicians) and health status variables (social role function, psychological well-being, energy, health distress and self-rated health).

The aim of the current study was to determine the extent to which health care utilization (physician visits, ED visits, hospitalizations) as measured by administrative data claims was reduced following the tele-CDSMP. Specifically, the objectives were 1) to describe patterns of health care utilization (number of physician visits, ED visits and hospitalizations) 6, 12 and 18 months prior to participating in the tele-CDSMP with the same time intervals following initial participation; and 2) to examine the extent to which health care utilization was reduced in the 12-month period following completion of the tele-CDSMP compared to the 12-month period prior to the program.

## Methods

### Design

Retrospective cohort study.

### Data sources

#### Tele- CDSMP participants

In the tele-CDSMP study, participants (n = 213) from 13 northern Ontario communities were enrolled in 19 six-week CDSMP courses between September 2007 and June 2008 with follow-up evaluations at 4 months and at 12 months [15]. Participants had a self-reported physician diagnosis of chronic lung disease, heart disease, stroke or chronic arthritis, were required to speak and read English and be able to attend a 2-hour session, once a week for 6 weeks. One hundred and six participants completed the follow up evaluation at 12 months.

#### Variables collected in the tele- CDSMP study

In the tele-CDSMP, we collected by telephone a number of outcome measures developed and validated by the Stanford Patient Education Centre [16]. Demographic information including age, sex, marital status, employment status, income, education level, and number and type of chronic conditions were collected at baseline for all participants. Measures of self-efficacy, health behaviors, and health status were collected at baseline, 4 months and 12 months [17]. The four health-related behaviors measured were: number of minutes per week each of stretching and strengthening exercise or aerobic exercise, use of cognitive symptom management techniques (mean of six items on a 5-point Likert scale), and use of techniques to improve communication with health care providers (mean of three items on a 5-point Likert scale) [16]. Seven validated health status measures were collected, including self-rated health status, the Stanford HAQ disability scale (8 items), pain (5 items), fatigue (5 items), social/role activity limitation (4 items), health distress (4 items), and psychological well-being (5 items) [18].

#### Recruitment for the current study

Approximately 1–2 years following enrollment in the tele-CDSMP, the original study interviewer contacted participants to obtain their health card number (HCN) and permission to use it for linkage to health administrative databases in Ontario. Of the 164 participants who completed follow up evaluations at 12 months, 106 individuals provided HCNs by telephone or mail (27 participants refused, 3 died, 9 moved and 19 were unreachable). Verbal and written consent were obtained for follow up evaluations with data linkage. This study was approved by the Research Ethics Boards at Women’s College Hospital and Sunnybrook Health Sciences Centre.

#### Administrative data sources and variables

To obtain data on health care utilization, the tele-CDSMP dataset with HCNs (a unique identifier) was linked to several administrative databases housed at the Institute for Clinical Evaluative Sciences (ICES). The Ontario Health Insurance Plan database contains all physicians’ fee-for-service claims. It contains information about each service provided by each physician, including the date, diagnosis, fee code, amount paid, HCN, and physician unique identifying number. The ICES Physician Database provides information on physician demographics and specialty training for those practicing in Ontario. The National Ambulatory Care Resource System database provides information on all visits to emergency departments; the main data elements are visit date and reasons for the ED visit defined by International Classification Diagnostic Codes-10th Canadian Version codes (ICD-10-CA). The Discharge Abstract Database provides information on hospital admissions. The health care utilization variables created from these administrative data sources were hospitalizations, all physician visits, general practitioners visits only, visits to specialist physicians and all ED visits.

### Statistical analyses

Descriptive statistics were computed for all health care utilization outcomes (physician visits, ED visits, and hospitalizations) and covariates. Health care utilization, including physician visits, ED visits and hospitalizations were compared for participants during the following time periods (6 months, 12 months and 18 months) prior to and after initial participation in the CDSMP using paired t-tests and Wilcoxon sign rank tests based on the distribution of data. In order to account for overall system and age-related changes in health care utilization, CDSMP participants were matched 5:1 to population controls on age, sex, postal code, and previous physician visits in the year prior to the program.

To examine the impact of the tele-CDSMP on health care utilization during the 12 months following the start of the program, adjusting for covariates, a block modeling approach was used to determine the covariates for final models [19]. Since outcomes consisted of counts, Poisson regression was used to adjust for covariates. Based on previous literature [2], subgroups of variables measured at four months (self-efficacy, health behaviors, and health status) and baseline (demographics, co-morbidities) were entered as separate blocks into models with each of physician visits, ED visits or hospitalizations as the dependent variable [19]. Except for socio-demographics, variables representing self-efficacy, health behaviors and health status measured at four months were chosen for modeling instead of those at baseline because it was hypothesized that participation in the tele-CDSMP would improve self-efficacy, health behaviors and health status and in turn reduce future health care utilization. For each covariate in the block model, we examined the odds ratio and p values. In an effort to be more inclusive, variables from each block that had a p value of less than 0.1 in at least one of the models (i.e., overall physician visits, GP/FP visits, ED visits, or specialist visits) were carried forward to be included in the determination of the final models [19].

To obtain the final models, Poisson Generalized Estimating Equations (GEE) longitudinal regressions were run to measure the change in health care utilization from pre- to post-program [20,21]. We included a parameter for overdispersion such that the variances were corrected in our models. The models included outcome data from two time periods for each participant (pre-program and post-program) along with baseline and time dependent covariates identified from the block models (sex, marital status, aerobic exercise score, cognitive symptom management, self-rated health). Outcomes consisted of the number of total visits of each type in both the 12 months prior to the program and in the 12 months following program participation (overall physician visits, GP/FP visits, specialist visits, and ED visits). A binary covariate, which was set to value one for post-program and zero for pre-program, was included in each model to estimate the adjusted effect of the program – the change in health care utilization between the 12 month period after the program and the 12 month period before. Interactions were also explored among confounders such as age, marital status and income level. Stratified models were constructed based on the interactions identified.

## Results

Of the 106 participants who provided permission for data linkage, only one could not be matched to the HCN provided. Of the remaining 105 individuals, one person had a large number of ED visits over the 18 month period. Reasons for visits were not related to chronic disease so it was decided that this outlier should be excluded leaving a total sample of 104 for analysis. The mean age of the linked cohort was 65.3 years and over three-quarters were female. The average number of chronic conditions was 2.2 and over 80% of the cohort had arthritis, 54.8% heart disease and 37.5% lung disease.

Using the self-report data we compared the tele-CDSMP participants with linked administrative data for health care utilization to the non-respondents (Table 1). There were no statistically significant differences between the groups for self-efficacy or among any of the health behavior or health status variables. However, participants were significantly younger, had higher incomes, and were also more likely to be married compared to non-respondents.

**Table 1:** Comparison of linked tele-CDSMP cohort versus non-linked

**Variables measured at baseline**		**Overall n (%) or mean (SD)**	
		**Linked**	Non-linked**
		**(n = 104)**	**(n = 107)**
Age*		65.3 (8.9)	68.4 (9.8)
Gender			
	Female	80 (76.9%)	80 (74.8%)
	Male	24 (23.1%)	27 (25.2%)
Marital Status*			
	Married	58 (55.8%)	48 (44.9%)
	Single	6 (5.8%)	8 (7.5%)
	Separated	4 (3.8%)	1 (0.9%)
	Divorced	9 (8.7%)	16 (15.0%)
	Widowed	27 (26.0%)	34 (31.8%)
Income*^+^			
	< 20,000	21 (20.2%)	24 (22.4%)
	20 – 39,000	32 (30.8%)	39 (36.4%
	40 – 59,000	32 (30.5%)	16 (15.0%)
	60 – 79,000	5 (4.8%)	9 (8.4%)
	≥ 80,000	9 (8.7%)	4 (3.7%)
Education			
	Less than high school	34 (32.4%)	42 (39.3%)
	High school diploma	33 (31.7%)	34 (31.8%)
	Post-secondary degree	32 (30.8%)	28 (26.2%)
	Graduate degree	5 (4.8%)	3 (2.8%)
Co-morbidity			
	Lung disease	39 (37.5%)	28 (26.2%)
	Heart disease	57 (54.8%)	50 (46.2%)
	Arthritis	87 (83.7%)	73 (68.2%)
	Stroke	13 (12.5%)	6 (0.06%)
	Diabetes	33 (31.7%)	26 (24.3%)
			
Self-efficacy score		6.5 (1.7)	6.7 (1.8)
			
Health Behaviours			
	Aerobic exercise behaviour (min/week)	127.6 (116.1)	136.1 (114.2)
	Stretching exercise behaviour (min/week)	55.4 (67.9)	47.7 (59.6)
	Cognitive symptom management score	1.7 (0.9)	1.6 (1.0)
	Communication with physicians score	3.2 (1.2)	3.1 (1.6)
Health Status			
	Self-rated health	3.3 (1.1)	3.3 (1.1)
	Stanford HAQ disability scale	0.4 (0.4)	0.4 (0.4)
	Social/Role Activities Limitation score	1.4 (1.1)	1.3 (1.1)
	Pain Severity	66.0 (18.5)	64.5 (20.8)
	Mood Assessment Scale	8.1 (6.1)	7.9 (5.9)
	Health distress	1.6 (1.2)	1.8 (1.3)

*Significant differences between linked cohort and non-linked cohort (p < 0.05).

**Participants were unable to be linked to the administrative data.

^+^Missing data due to prefer not to say and/or left blank.

The median number of total physician visits among the tele-CDSMP linked cohort 6, 12 and 18 months prior to the program were 4 (IQR = 2-7), 8 (IQR = 5-12), and 12 (IQR = 8-18), compared to 4 (IQR = 2–7), 8 (IQR 5–13) and 12 (IQR 8–18) post program, respectively (see Table 2). The median number of ED visits 6, 12 and 18 months prior to the program were 0 (IQR = 0-2), 1 (IQR = 0-3), and 2 (IQR = 0-4), compared to 0 (IQR = 0-1), 1 (IQR = 0-3) and 1 (IQR = 0-5) post program, respectively. Overall, there were no statistically significant differences pre and post program in any of the health care utilization variables at 6, 12 or 18 months. Compared to the matched general population, the CDSMP participants had significantly higher health care use.

**Table 2:** Health care utilization 6, 12 and 18 months prior to and 6, 12 and 18 months after participation in a tele-CDSMP, as measured by administrative data

	**Health care utilization visits overall sample**					
**Type of visit**	**Previous 6 months**	**Post 6 months**	**Previous 12 months**	**Post 12 months**	**Previous 18 months**	**Post 18 months**
*All physician*						
Mean (SD)	4.8 (4.1)	4.9 (3.9)	9.3 (6.9)	9.7 (6.9)	13.5 (9.5)	14.1 (9.7)
Median (IQR)	4 (2–7)	4 (2–7)	8 (5–12)	8 (5–13)	12 (8–18)	12 (8–18)
*General practitioner*						
Mean (SD)	3.3 (3.0)	3.2 (2.6)	6.5 (5.1)	6.4 (5.1)	9.4 (7.1)	9.3 (7.2)
Median (IQR)	3 (1–5)	3 (1–4)	5 (3–9)	6 (3–8)	8 (5–11)	8 (5–12)
*Specialist*						
Mean (SD)	1.5 (2.0)	1.7 (2.4)	2.8 (3.3)	3.3 (3.5)	4.1 (4.6)	4.8 (4.8)
Median (IQR)	1 (0–2)	1 (0–3)	2 (1–4)	2 (1–5)	3 (1–5)	3 (1–7)
*Emergency*						
Mean (SD)	1.3 (2.9)	1.0 (1.7)	2.2 (3.5)	2.0 (2.8)	3.2 (4.9)	3.1 (4.4)
Median (IQR)	0 (0–2)	0 (0–1)	1 (0–3)	1 (0–3)	2 (0–4)	1 (0–5)
*Hospitalizations*						
Mean (SD)	0.1 (0.4)	0.2 (0.4)	0.1 (0.5)	0.3 (0.7)	0.3 (0.5)	0.4 (0.8)
Median (IQR)	0 (0–0)	0 (0–0)	0 (0–0)	0 (0–0)	0 (0–0)	0 (0–0)

In the block models for covariate selection, sex, marital status, aerobic exercise score, cognitive symptom management, and self-rated health were identified as significant covariates to be included in the final GEE models (see Table 3). In the GEE models, an interaction effect was identified with age. Thus models were stratified by age based on the median (≤66 and > 66 years).

**Table 3:** Variables selected from block modeling

	**Physician visits**		**GP/FP visits**		**Specialist visits**		**ED visits**	
	**OR (95% CI)**	**p-value**	**OR (95% CI)**	**p-value**	**OR (95% CI)**	**p-value**	**OR (95% CI)**	**p-value**
*Age*	1.0 (1.0- 1.0)	0.1	1.0 (1.0-1.0)	0.1	1.0 (1.0-1.0)	0.5	1.0 (0.9-1.0)	0.09
*Sex*								
Female	Reference		Reference		Reference		Reference	
Male	1.6 (1.1-2.3)	0.1	0.1 (1.0-1.0)	0.09	1.0 (1.0-1.0)	0.48	1.0 (0.9-1.0)	0.09
*Marital Status*								
Married	Reference		Reference		Reference		Reference	
Single	0.6 (0.3-1.5)	0.3	0.5 (0.2-1.5)	0.2	0.8 (0.3-2.6)	0.7	0.09 (0.003-2.6)	0.2
Separated	0.8 (0.4-1.7)	0.6	0.6 (0.2-1.6)	0.3	1.2 (0.5-3.1)	0.7	1.4 (0.6-3.6)	0.5
Divorced	1.3 (0.8-2.1)	0.4	1.4 (0.8-2.4)	0.3	1.1 (0.5-2.5)	0.8	0.7 (0.3-2.0)	0.5
Widowed	1.4 (1.0-2.0)	0.08	1.6 (1.0-2.4)	0.03	1.1 (0.6-2.0)	0.7	1.2 (0.6-2.4)	0.6
*Aerobic Exercise Score*	1.0 (1.0-1.0)	0.007	1.0 (1.0-1.0)	0.05	1.0 (1.0-1.0)	0.02	1.0 (1.0-1.0)	0.008
*Cognitive Symptom Management*	0.9 (0.8-1.0)	.08	0.9 (0.8-1.1)	0.3	0.8 (0.6-1.0)	0.05	1.2 (0.9-1.6)	0.2
*Self-rated Health*	1.0 (0.9-1.3)	0.6	1.0 (0.8-1.3)	0.8	1.1 (0.8-1.5)	0.6	1.6 (1.1-2.3)	0.02

Table 4 shows the health care use patterns for individuals ≤ 66 years of age compared with those > 66 years. For those individuals ≤ 66 years, there was a 34% increase in physician visits one year post program (OR 1.3, 95% CI 1.1-1.6, p = 0.003), and no significant difference in ED visits (OR 1.1, 95% CI 0.8-1.6, p > 0.05; see Table 5). The tele-CDSMP did not have a significant impact on physician visits for individuals > 66 years; however, there was a trend towards significance with a 41% reduction in ED visits during the year following the program (p = 0.08).

**Table 4:** Health care utilization 6, 12 and 18 months prior to and 6, 12 and 18 months after participation in a tele-CDSMP stratified by age

	**Mean (SD) health care utilization visits**					
**Variable**	**Previous 6 months**	**Post 6 months**	**Previous 12 months**	**Post 12 months**	**Previous 18 months**	**Post 18 Months**
All Physician visits						
<= 66	4.8 (3.7)	5.3 (4.1)	9.2 (6.0)	10.2 (7.2)	12.9 (8.4)	14.8 (9.7)
> 66	4.9 (4.5)	4.6 (3.8)	9.4 (7.7)	9.1 (6.7)	14.1 (10.5)	13.3 (9.8)
GP/FP visits						
<= 66	3.1 (2.7)	3.3 (2.6)	6.1 (4.8)	6.5 (5.2)	8.9 (7.0)	9.6 (7.4)
> 66	3.5 (3.3)	3.1 (2.5)	6.8 (5.5)	6.2 (5.0)	9.9 (7.3)	8.9 (7.1)
Specialist visits						
<= 66	1.7 (2.0)	2.0 (2.5)	3.1 (3.2)	3.6 (3.6)	4.1 (3.8)	5.2 (4.8)
>66	1.3 (2.1)	1.5 (2.3)	2.5 (3.5)	2.9 (3.3)	4.1 (5.2)	4.3 (4.8)
ED visits						
<= 66	0.9 (1.9)	1.3 (2.1)	2.0 (3.3)	2.2 (3.2)	3.2 (5.0)	3.4 (4.7)
> 66	1.7 (3.7)	0.8 (1.6)	2.3 (3.7)	1.7 (2.3)	3.1 (4.8)	2.9 (4.2)
Hospitalizations						
<= 66	0.1 (0.5)	0.2 (0.4)	0.1 (0.5)	0.3 (0.6)	0.2 (0.5)	0.4 (0.7)
> 66	0.1 (0.4)	0.1 (0.4)	0.2 (0.5)	0.3 (0.8)	0.3 (0.5)	0.4 (0.9)

**Table 5:** The Impact of the tele-health program on health care utilization using generalized estimating equation models adjusting for covariates

	**Age ≤ 66**			**Age > 66**		
	**OR**	**95% CI**	**p-value**	**OR**	**95% CI**	**p-value**
Physician Visits*	1.3	1.1- 1.6	0.003	0.9	0.7-1.2	0.6
GP/FP Visits	1.3	1.0-1.6	0.02	0.9	0.7-1.1	0.2
Specialist Visits	1.5	1.1-2.0	0.006	1.04	0.6-1.7	0.3
ED visits*	1.1	0.8-1.6	0.6	0.6	0.3-1.7	0.08

*adjusted for sex, marital status, aerobic exercise score, cognitive symptom management, self-rated health.

## Discussion

Our results are consistent with the recent meta-analysis of evaluations of the CDSMP by Brady and colleagues [11], as in our unadjusted analyses we found no significant differences for visits to physicians, EDs or hospitalizations using administrative data before versus after participation in the tele-CDSMP. Based on the interaction with age, further exploration with a larger sample is needed. After adjustment for covariates, we found that the younger group with chronic conditions (age 66 years or less) had a statistically significant increase in physician visits post intervention, while for the older group (>66 years) there was a trend towards reduced ED visits 12 months after participation in the tele-CDSMP. A possible explanation is that the CDSMP encourages participants to assume a more active role in managing their health condition, and that ‘management’ is construed as consultation with health care practitioners. The CDSMP is consistent with the shift to a more “patient-centered” approach to health care, where provider-patient relations change from being ‘directive’ to ‘shared’ through joint definition of problems, treatment goals and management strategies, which may result in more or better quality visits [22-25]. Also, in countries with universal health care there may be less incentive for patients to reduce the number of visits. For example, those with more common chronic conditions who have a steady pattern of service use may find little room, or need, for change especially when this is linked to codes of professional practice or payment on the supply side. While there is universal access to health care in Ontario, physician payment is based on fee-for-service. Structured physician visits may be particularly true for the older patients with chronic conditions as they tend to have regularly scheduled follow-up visits for example for medication refills or blood pressure checks and hence no differences in physician visits were seen for this age group in our study. The trend toward decreased ED visit use among the older age group needs to be confirmed in future studies. It could be postulated that the CDSMP has had the desired effect and taught these participants how better to manage and cope with their condition and thus reduce the need for visits to the emergency department. This is supported by the fact that in the main study evaluating the tele-CDSMP, as well as in the current study, significant improvements in self-efficacy, health behaviors and health status were observed after participation in the program [15].

Recently, Hamar and colleagues examined the impact of a chronic disease management program on hospital admissions and readmissions for persons with heart disease or diabetes in Australia [26]. The intervention consisted of personalized online health support, involving health assessments, health action plans, education, and health behavior tracking for a period of 18 months. In addition, nurses provided phone calls to participants who had been identified as needing more intensive support. This study showed decreased hospitalizations during the 12 and 18 month period for participants while enrolled in the program for both the diabetes and heart disease cohort, with the latter group also showing significant reductions with respect to readmissions. While we did not find significant reductions in hospitalizations post tele-CDSMP, there are some important and relevant differences to highlight. Participants in Hamar et al.’s study [26] had higher baseline hospitalization rates compared to our cohort and also comprised a larger proportion of persons with heart disease. Compared to the Stanford CDSMP, the intervention was more intensive and comprehensive in that it targeted vulnerable individuals who might have needed more support with additional follow-up calls from nurses.

Strengths and limitations of this study are noted below. The main strength of this study was the use of administrative data to determine health care utilization, which is not subject to recall bias. A study of seniors in Ontario examining agreement between self-reported and routinely collected health-care utilization data has shown both over and under-reporting of self-reported use of health services [27]. Another strength of the study was the use of GEE to examine the impact of the CDSMP, as previous studies on health care use relied on descriptive statistics. Using GEE analysis, we are able to account for clustered observations. While our study has generated some interesting hypotheses for future research the findings need to be confirmed,and the limitations highlighted. The study participants were from rural and remote communities in Canada and even though there is universal access to health care, the availability of services in these regions, e.g. access to specialists, is less than in urban centers [28] which may influence patterns of health care utilization. Another limitation is the small sample size for the multivariate regression with a stratified sample, perhaps due to the fact that individuals were being re-contacted one year after they participated in the initial study. The respondents were slightly younger and of higher income than the group who did not consent, which may influence the observed pattern of health care utilization and thus the generalizability of results to all older rural populations, since higher income is associated with lower health care utilization [29]. Additionally, differences in ED visits by age group were identified using post-hoc analyses and needs to be confirmed in future studies. Finally, we do not know the length of time since chronic disease diagnosis.

## Conclusions

In summary, this is the first study to examine health care use following participation in the CDSMP in a Canadian population and to use administrative data to measure health care utilization. Similar to other studies that used self-report measures to evaluate health care use we found no differences in health care utilization before and after participation in the CDSMP. Future research needs to confirm our findings and examine the impact of the CDSMP on health care utilization in different age groups to help to determine whether these interventions are more effective with select population groups.

## Competing interests

The authors declare that they have no competing interests.

## Authors’ contributions

SJ took primary responsibility for the design of the study, the acquisition, analysis and interpretation of data, and drafting and revising the article. She gave final approval of the version to be published. She accepts full responsibility for the work and the conduct of the study, had access to the data, and controlled the decision to publish. SG took primary responsibility for the design of the study, the acquisition, analysis and interpretation of data, and drafting and revising the article. GH, NS, and MZ provided advice and direction for the study design and contributed to data interpretation. They revised the article critically for important intellectual content. WL provided advice and direction for the study design, statistical analyses and contributed to data interpretation. She revised the article critically for important intellectual content. MM provided advice and direction for the study design and contributed to data analysis and interpretation. He revised the article critically for important intellectual content. All authors read and approved the manuscript.

## Pre-publication history

The pre-publication history for this paper can be accessed here:

http://www.biomedcentral.com/1472-6963/14/198/prepub
